# Analytic descriptions of parabolic X-ray mirrors

**DOI:** 10.1107/S1600577522004593

**Published:** 2022-05-25

**Authors:** Kenneth A. Goldberg

**Affiliations:** a Advanced Light Source, Lawrence Berkeley National Laboratory, 1 Cyclotron Road, Berkeley, CA 94720, USA

**Keywords:** X-ray, mirror, parabolic, collimator, focusing

## Abstract

On X-ray beamlines and telescopes, glancing-incidence mirrors with parabolic profiles are used to collimate, focus, and collect light. Here, analytic descriptions for paraboloidal, plane-parabolic, and parabolic cylindrical mirrors in several congruent geometries that are commonly used in fabrication, metrology, and modeling are provided. The exact expressions are derived directly from Fermat’s principle, without coordinate transformations, in several mirror-centered coordinate systems, including one with the surface tangent to the central point of intersection. Coefficients for a sixth-order polynomial series approximation are calculated for that coordinate system.

## Abstract 2

Exact analytic descriptions of parabolic, paraboloidal, and parabolic cylindrical X-ray mirrors in a mirror-centered coordinate system for design, modeling, fabrication, and testing are given.

## 1. Introduction

Parabolic surfaces are used in a wide variety of light-concentrating and light-collimating applications, across the electromagnetic spectrum, from satellite dishes to automobile headlamps. The properties of parabolas as conic sections have been studied since antiquity by scholars including Menaechmus, Apollonius, Pappus, and Archimedes. Legend states that to protect Syracuse from Roman ships, Archimedes arranged 60 men armed with ‘burning mirrors’ to set ships ablaze with focused sunlight (Valiullin & Tarabarin, 2010 ▸).

Parabolic mirror shapes have long been used at glancing angles of incidence in Wolter-type X-ray telescope designs (VanSpeybroeck & Chase, 1972 ▸). For more than 40 years, concave parabolic mirrors have provided collimation of diverging X-ray beams and focusing of collimated beams (Eberhardt *et al.*, 1978 ▸; MacDowell *et al.*, 2004 ▸). Such mirrors are essential optical elements for beamlines with monochromators that operate in collimated beams, such as double-crystal monochromators.

Parabolic mirror profiles can be achieved through mechanical bending (Underwood & Turner, 1977 ▸; Johnson, 1982 ▸; Heald, 1982 ▸; Franks *et al.*, 2000 ▸), and they can be fabricated in their natural shape with relatively high quality (Thiess *et al.*, 2010 ▸). Their focusing properties have been studied theoretically, with ray and wave optics, and experimentally (Raimondi & Spiga, 2010 ▸). Refractive parabolic lenses are also in use (Lengeler *et al.*, 2002 ▸) with hard X-ray beams.

In this article, we describe three classes of mirror shapes all with parabolic tangential profiles. (1) Paraboloidal mirrors are surfaces of revolution defined by a generating parabola rotating about its axis of symmetry. They collimate a point source to a plane wave or vice versa. (2) Plane-parabolic mirrors are uniform (*i.e.* flat) in the sagittal direction, and collimiate (or focus) only in one direction. (3) Parabolic cylinders have a uniform sagittal radius of curvature along their tangential length allowing them to simultaneously focus and collimate in orthogonal directions. However, their sagittal focusing suffers from aberrations for non-paraxial rays. Diaboloid mirrors are ideal surfaces with parabolic tangential profiles that simultaneously collimate and focus without aberrations. They are described by Yashchuk *et al.* (2021 ▸) and Sanchez del Rio *et al.* (2021 ▸).

Parabolic curves in a plane are fundamental shapes that are easily described with second-order equations and simplify in a coordinate system where one axis runs parallel to the axis of symmetry. However, in arbitrary arrangements, their description is somewhat more complex.

We seek convenient solutions with two or three parameters: the distance to focus, the glancing angle of incidence at the central point of intersection, and, in the cylindrical case, the distance to the horizontal source point. Such descriptions avoid cumbersome coordinate transformations and are most convenient for fabrication, testing, and modeling in X-ray optics. In particular, the coordinate system with the mirror surface tangent to the *xy* plane (*i.e.* zero slope at the center) is the most generally applicable for X-ray optical descriptions. Furthermore, all surface descriptions are given with the coordinate origin at the central point of intersection.

This approach addresses some of the key challenges in accurately and precisely modeling X-ray optics designed for wavefront preservation. Consider the fact that conjugate distances can be from meters to tens of meters or more; mirrors can be on the order of 1 m long in some cases, and several cm wide; and optical modeling requires sub-nm and sub-nrad surface shape accuracy. Thus, numerical calculations must accommodate precision far exceeding 11 orders of magnitude. In such cases, we find that polynomial series approximations are challenged by mirrors that are long or wide. Empirical surface mesh solutions (*e.g.* for ray tracing), which can be transformed into arbitrary positions, must contain a high density of points, reducing calculation efficiency. Having closed-form single expressions for the mirror surfaces enables efficient description and modeling with arbitrary precision.

### 1.1. Conventional parabola description and definitions

**Figure 1:**
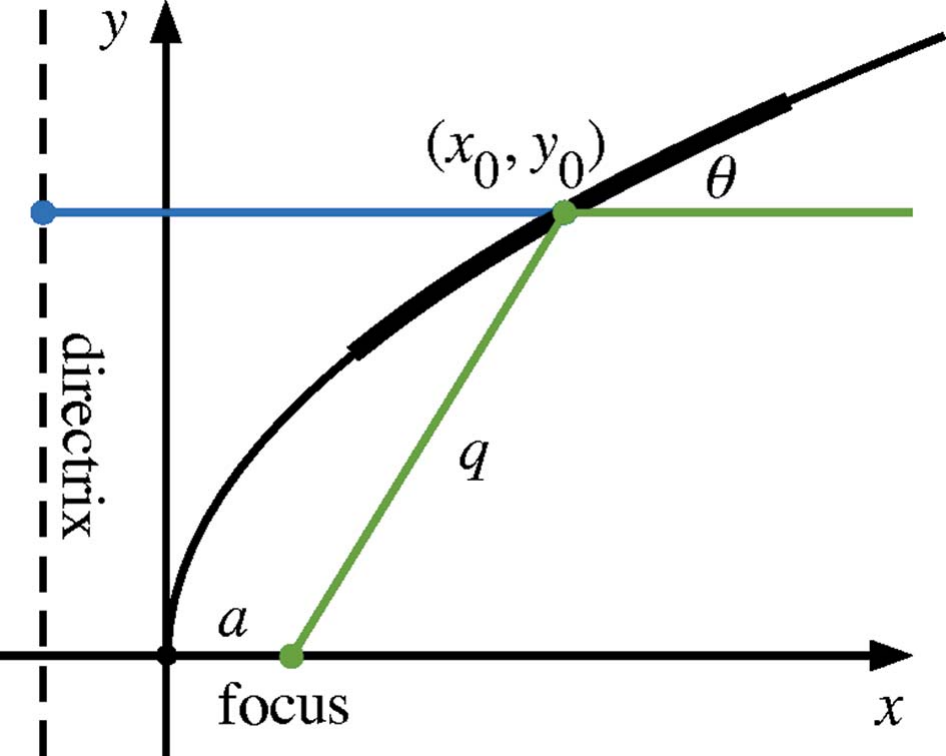
A section of a parabola showing the focus and the directrix line, and a ray reflected from the surface (green). The *x*-axis becomes the axis of rotation for a paraboloid.

There are several available definitions of the parabola. In a mathematically simple form, the parabola is the locus of points in a plane that are equidistant from both the directrix and the focus. Oriented in the *xy* plane with the axis of symmetry along the *x*-axis (Fig. 1 ▸), with the focus at (0, *a*), and the directrix at *x* = −*a*, the equation for this shape is 

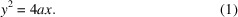

In optics, however, the most important *functional* definition is that all incident rays parallel to the axis of symmetry are reflected toward the focus (*e.g.* the green line in Fig. 1 ▸). Conversely, all rays leaving the focus are reflected parallel to the axis of symmetry. Following West & Padmore (1987 ▸), Peatman (1997 ▸), and Takei *et al.* (2016 ▸), we find 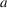 = 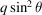 , where *q* is the distance from the focus to the center of a mirror segment (as represented in Fig. 1 ▸), and θ is the glancing angle. In this coordinate system, the intersection point is 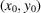 = 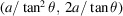 .^1^ Peatman provides coefficients for polynomial series expansion of the surface about the central point in the coordinate system with orthogonal axes tangent to and perpendicular to the center of the mirror. We address this surface approximation in Section 8.

Other helpful descriptions of parabolas are provided by Schuster *et al.* (1999 ▸) and Protopopov *et al.* (2000 ▸) who describe optical coatings for high X-ray reflectivities with varying incidence angles.

## 2. Three mirror geometries

**Figure 2:**
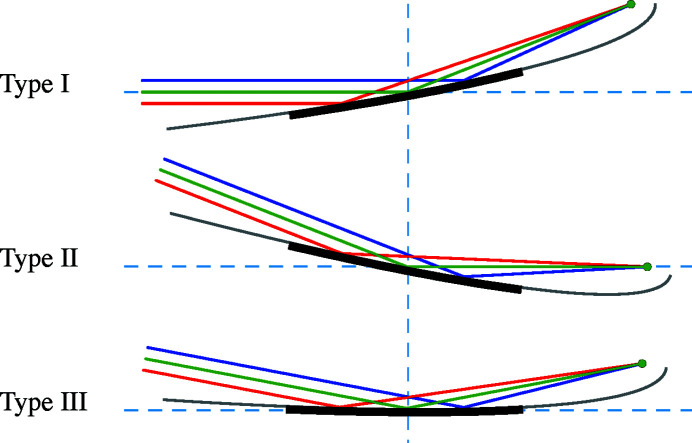
Three congruent descriptions of a concave, tangentially parabolic mirror geometry shown as a cross-section in the meridional plane (*x* = 0). The mirror is used to focus a collimated beam, or to collimate a divergent beam in one direction. The mirror (thick line) is a section of a parent parabolic curve (gray line).

We can describe parabolic surfaces in three typical glancing-incidence orientations, shown in Fig. 2 ▸. These descriptions are congruent, varying only in the orientation with respect to the coordinate axes. They simplify the surface description when the incident or reflected beams run along the *y*-axis, or when the center of the surface is tangent to the *xy* plane.

We describe six related geometries labeled as Types I, II, and III, and with orientations A and B. In each case, the concave reflective surface faces upward. We use a coordinate system that is common in glancing-incidence X-ray optics, with *x* as the sagittal coordinate, *y* as the tangential coordinate, in the general direction of propagation, and *z* as the surface height of the mirror. The surface of interest is *z*(*x*, *y*). The surfaces pass through the origin at the central point, so *z*(0, 0) = 0.

Type I paraboloids focus collimated light that is parallel to the *y*-axis. Type II paraboloids are illuminated with an off-axis collimated beam and focus light in the direction parallel to the *y*-axis. Type III paraboloids have zero slope at the central point of intersection: collimated light is incident and is reflected at an angle from the *y*-axis. The Type III description is most convenient for manufacture and metrology since the part has no tilt at the center and the overall tilt is minimized. The other, congruent descriptions may be more convenient for modeling because they describe the exact surface, in place, without rotation or translation.

Each of these shapes can be oriented with the input and output reversed. Type A indicates that incident collimated light is focused to a point at distance *q*; while Type B takes diverging light from a source at distance *p* and collimates it upon reflection. For example, in an X-ray beamline built around a double-crystal monochromator, we might select a Type IIB plane-paraboloid as the first element, vertically collimating light from a point source, and a Type IA paraboloid or parabolic cylinder after the monochromator to focus the collimated light in two directions to a common point. Of course, with matching *p* or *q* and θ, two related surface descriptions are congruent: it is only the mathematical descriptions that vary to facilitate modeling or manufacture.

## 3. Applying Fermat’s principle

In geometrical optics, descriptions of optimal surfaces follow from Fermat’s principle of least time, where all ray paths through an optical system have equivalent length. In our case, the surface reflection breaks the ray paths into two segments; the total path lengths are computed from their sum. For convenience, we describe the surfaces as passing through the origin. Furthermore, we neglect phase changes upon reflection, which may be angle- and polarization-dependent in some cases.

In the following sections, mathematical derivations are made for the Type A cases. Type B cases follow from simple substitution. We can describe the path lengths of the incident collimated rays using the distance from an inclined plane or line source, upstream of the mirror. In the solution, the distance to this source is arbitrary and falls out of the calculation, so it simplifies the derivations to pass this source line or plane through the origin, with no loss of generality. In this position, distances from points on the surface to the source may be positive or negative, and the sign must be preserved in the total-ray-distance calculation.

The reflected light is focused to a distance *q* relative to the center of the mirror. In all cases, θ is the central, glancing angle of incidence.

The following derivations are made for paraboloids. Plane-parabolic mirrors follow the same equations with the *x* term set to zero. Cylindrical parabolic mirrors are defined from the plane-parabolic shape with a uniform sagittal radius, as discussed in Section 7.

## 4. Type I paraboloid derivation

**Figure 3:**
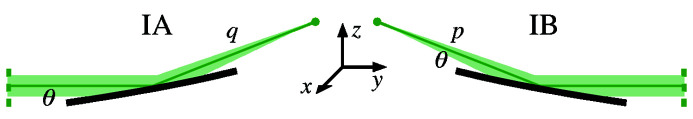
The Type IA paraboloids focus a collimated beam incident parallel to the *y*-axis. Type IB collimates light from an off-axis point source at distance *p*.

The central ray is incident parallel to the *y*-axis from a source in an *xz* plane. With an angle of incidence θ, the reflected ray is inclined at 2θ from the axis. For Type I paraboloids, light is focused either to a point 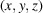 = 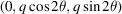 or from a point 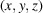 = 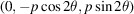 as shown in Fig. 3 ▸. The statement of Fermat’s principle in the Type IA case is the sum of the two ray segments, 

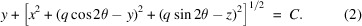

At the origin, *x* = *y* = *z* = 0, thus *C* = *q*.

The solution proceeds by isolating the square root and squaring both sides. Grouping terms with respect to powers of *z*, the intermediate equation is 

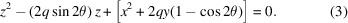

We can solve for *z* using the quadratic equation. Of the two solutions, we take the negative root to obtain the upward-facing, concave surface that passes through the origin, 

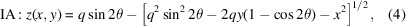




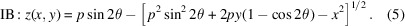

Equation (5) comes from equation (4) with the substitutions *q* → *p* and *y* → −*y*.

## 5. Type II paraboloid derivation

**Figure 4:**
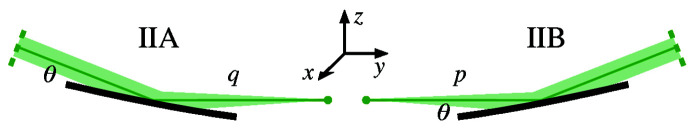
The Type IIA paraboloid focuses a collimated beam to a distance *q* with the reflected central ray parallel to the *y*-axis. Type IIB collimates light from a point source at distance *p* with the incident ray along the *y*-axis.

In the Type IIA case, collimated light is incident from above the *y*-axis, and is reflected in the direction parallel to the *y*-axis. Light is focused to a point (*x*, *y*, *z*) = (0, *q*, 0), as shown in Fig. 4 ▸. The source plane from which the rays emanate has normal 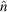 = 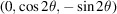 , inclined downward. In the Type IIB case, diverging light from a point source at (*x*, *y*, *z*) = (0, − *p*, 0) is centered about the *y*-axis and is collimated in an upward direction, inclined by 2θ from the *y*-axis.

In the Type IIA case, the distance from the source plane to a point on the mirror surface is 

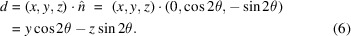

Here, the calculation simplifies when we allow the source plane to pass through the origin and allow negative distances in the ray path lengths. If an arbitrary source distance were applied, that source position would ultimate fall out of the calculation.

The statement of Fermat’s principle for the Type IIA case is the constant sum of the two ray segments, 

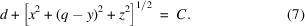

Again, the mirror surface passes through the origin, where *x* = *y* = *z* = 0, thus *C* = *q*. Equation (7) can be written as 

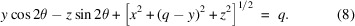

As in Section 4 above, we isolate the square root and square both sides. Then the powers of *z* are grouped and the second-order equation is solved using the quadratic equation. The solutions are 

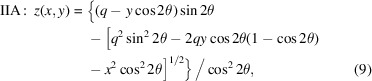




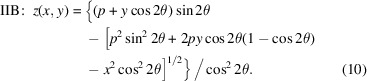

Equation (10) arises from equation (9) with the substitutions *p* → *q* and *y* → −*y*.

## 6. Type III paraboloid derivation

**Figure 5:**
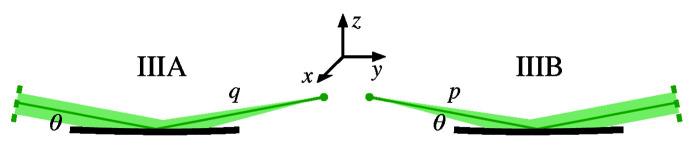
The Type III plane-parabolas are aligned with the surface tangent to the *y*-axis at the central point of intersection. This is the coordinate system most convenient for fabrication and metrology.

In the Type III descriptions, the surface is tangent to the *xy* plane at the central point of intersection. This is the most common, mirror-centered coordinate system description for X-ray mirrors in modeling, fabrication, and metrology. For Type IIIA, collimated light is incident from above with glancing angle θ, as shown in Fig. 5 ▸. The light is reflected upward toward the focal point at distance *q*, with 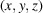 = 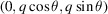 . Similarly for Type IIIB, incident diverging light from a source at distance *p* is collimated as a beam inclined upward.

In the Type IIIA description, the arbitrarily placed source plane, from which the rays emanate, has a surface normal 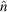 = 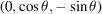 , inclined downward. To simplify the calculation, as before, we take this plane to pass through the origin. The distance from a point on the mirror surface to the source plane is 

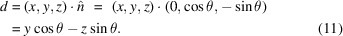

The statement of Fermat’s principle is this distance plus the distance to focus,

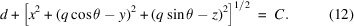

With *x* = *y* = *z* = 0, we find that *C* = *q*. Once again, we isolate the square root and square both sides, group powers of *z* and apply the quadratic equation,

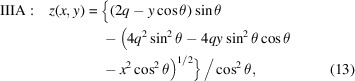




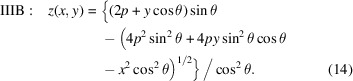




## 7. Parabolic cylinder mirrors

For narrow ray bundles, focusing in the sagittal *x* direction can be performed with cylindrical or toroidal mirrors which have a uniform, concave, circular cross section in the sagittal direction. Such elements can be mechanically bent along the tangential direction to have a parabolic profile in the *yz* plane (Franks *et al.*, 2000 ▸).

Coddington’s equation (Kingslake, 1994 ▸) provides the optimal sagittal radius for paraxial rays, based on the *p* and *q* distances and the glancing angle of incidence, θ,

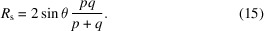

When the incident light is collimated, *p* → ∞, and 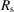 = 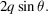 Similarly, when diverging light is collimated, *q* → ∞, and 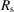 = 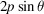 .

Since the object or image distance varies along the part, the *ideal* shape has a tangentially varying sagittal radius [as in equations (13) and (14)]. The recently described diaboloid shape satisfies this ideal focusing condition (Yashchuk *et al.*, 2021 ▸). Generally speaking, fabrication is simplified with a uniform sagittal radius.

To mathematically describe parabolic cylinder mirrors, we first extract the central parabolic shape of the Type III paraboloids from equations (13) and (14), in the meridional (*x* = 0) plane, and then add a uniform sagittal Δ*z*(*x*) term that includes a constant sagittal radius of curvature, *R*_s_, 

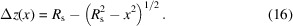

Combined, these become 

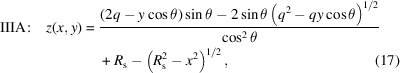




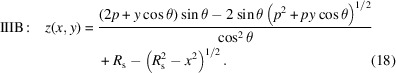




## 8. Series expansions

Many researchers and optical designers use polynomial series expansions about the mirror center to describe surface shapes. Here we present Maclauren series expansions that approximate the Type IIIA/IIIB paraboloids, and the parabolic cylindrical shapes. We compare fourth- and sixth-order expansions with the ideal surfaces.

A conventional series expansion used in X-ray optics takes the form 

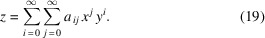

We define the center of the mirror to be zero height (*a*_00_ = 0). With zero tilt at the center, terms with *i* = 1 or *j* = 1 are equal to zero. Furthermore, symmetry about the *y*-axis dictates that all odd-ordered terms in *x* must be zero.

### 8.1. Paraboloid and plane-parabola approximation

For the Type IIIA paraboloid (focusing an incident collimated beam) the series expansion coefficients, up to (*i* + *j*) ≤ 6, are as follows. Symbolic calculations were made with verification in *Mathematica* (Wolfram Research Inc., 2020 ▸),

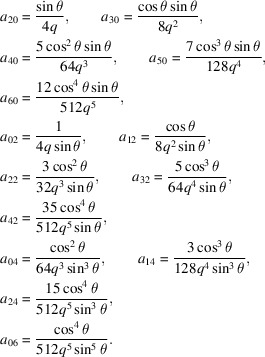

For Type IIIB paraboloids, substitute *q* → *p* in the above, and flip the sign on terms in odd powers of *y*: *a*_*ij*_ → −*a*_*ij*_ for odd *i*. For plane-parabolic surfaces, additionally set all coefficients of non-zero powers of *x* to zero (*i.e.* *a*_*ij*_ = 0 for all *j* > 0).

### 8.2. Paraboloid series approximation example

**Figure 6:**
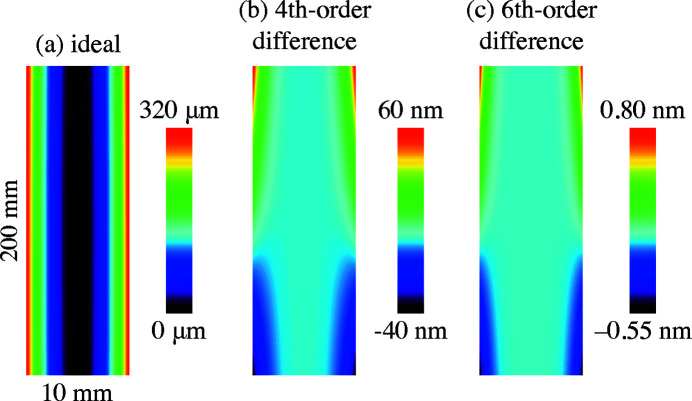
Comparison of the ideal surface to two polynomial expansions. (*a*) Ideal paraboloid, computed for *q* = 4 m, θ = 5 mrad. (*b*) Point-by-point comparison with a series expansion up to (*i* + *j*) ≤ 4. (*c*) Comparison with a series expansion up to (*i* + *j*) ≤ 6. Note that the rendering of the domain is not isometric.

Figure 6 ▸ compares the ideal paraboloid shape with two series approximations up to fourth order and sixth order, respectively, in the combined exponents. Comparison is made across a domain of |*x*| < 5 mm, and |*y*| < 100 mm, where the maximum surface height is 320.925 µm. The *q* and θ values are 4 m and 5 mrad, respectively. Relative to the ideal surface, the peak height error from the fourth-order approximation is 59.082 nm, and from the sixth-order approximation it is 0.770 pm. We observe that a fourth-order approximation may not be sufficient for demanding beamline mirror designs with nanometer tolerances. The sixth-order approximation has its largest error magnitude in the downstream corners, and so it would meet a fraction-of-a-nanometer tolerance requirement across a center-weighted beam footprint.

### 8.3. Parabolic cylinder approximation

The parabolic cylinder series expansion has no *xy* cross-terms because the variables are separable in equations (17) and (18). The *x* dependence is purely circular. The coefficients are as follows,

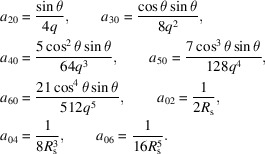

As above, a sixth-order expansion will be necessary to achieve nanometer tolerances in the sagittal direction, using the example geometry above.

## 9. Conclusion

Parabolic mirror shapes will continue to be used in X-ray optics, either individually or paired with other surfaces, in the most demanding applications. The most convenient descriptions of the parabolic mirror shapes may be different for modeling or manufacturing, depending mainly on the tilt of the part and the direction of the incoming or outgoing rays relative to the coordinate axes. Metrology and fabrication are simplified by surface descriptions that have zero slope at the center, minimizing both the peak tilt value and the net tilt across the surface. This configuration is labeled Type III in our discussion. In practice, *on beamlines*, parabolic mirrors are often used with either the collimated light or the focused light parallel to the general direction of propagation. Those configurations are labeled Type I and Type II, respectively. For each case, with different central tangential tilts, we have presented direct expressions for the surfaces that are not derived from coordinate transformations.

Since Type A mirror shapes with matching *q* values (or Type B mirror shapes with matching *p* values) are mutually congruent, corresponding equations for the three mirror orientation types *all describe the same surface*. This property facilitates the use of these equations in different modeling and metrology scenarios with confidence that the rotated surfaces are correct and will match from one use-case to another.

Comparison of the ideal shape with polynomial series approximations in a relevant design case shows that limiting a calculation to fourth order may result in a surface height error of tens of nanometers, while a sixth-order description can be accurate to tenths of a nanometer.

## Footnotes

1 Some sources take the incidence angle defined relative to the surface normal vector. Here we use the glancing angle.
